# Phenotypic Plasticity in Gut Length in the Planktivorous Filter-Feeding Silver Carp (*Hypophthalmichthys molitrix*)

**DOI:** 10.1100/tsw.2008.37

**Published:** 2008-02-19

**Authors:** Zhixin Ke, Xie Ping, Longgen Guo

**Affiliations:** Donghu Experimental Station of Lake Ecosystems, State Key Laboratory for Freshwater Ecology and Biotechnology of China, Institute of Hydrobiology, The Chinese Academy of Sciences, Wuhan 430072, China

**Keywords:** silver carp, relative gut length, resource polymorphisms, phenotypic plasticity, food resource, zooplankton

## Abstract

Phenotypic plasticity widely exists in the external morphology of animals as well as the internal traits of organs. In the present study, we studied the gut length plasticity of planktivorous filter-feeding silver carp under different food resources in large-net cage experiments in Meiliang Bay of Lake Taihu in 2004 and 2005. There was a significant difference in stocking density between these 2 years. Under a low stocking density and abundant food resources, silver carp increased their energy intake by feeding on more zooplankton. Meanwhile, silver carp adjusted their gut length to match the digestive requirements of food when exposed to different food resources. In the main growth seasons (from April to October), silver carp significantly increased their relative gut length when feeding on more phytoplankton in 2005 (*p* < 0.01, 9.23 ± 1.80 in 2004 and 10.77 ± 2.05 in 2005, respectively). There was a nearly significant negative correlation between zooplankton proportion in the diet and the relative gut length when silver carp were stocked in a high density (*p* = 0.112). It appears that silver carp might have evolved plasticity to change their gut length rapidly to facilitate efficient utilization of food resources. Such resource polymorphisms in the gut may be a good indication of temporal adaptation to resource conditions. Our work provided field evidence for understanding the functional basis of resource polymorphisms and the evolution of phenotypic plasticity in planktivorous filter-feeding fish.

## INTRODUCTION

Resource polymorphisms, a tight link between individual resource use and morphology, are widely observed in animal populations[1]. Many studies suggest that resource polymorphisms are an important first step in the process of sympatric speciation[2,3]. Resource polymorphisms have attracted considerable attention in recent years[4].

Environmental variation can induce dramatic changes in the traits of organisms and many of these phenotypically plastic changes appear to be adaptive[5]. These polymorphisms may also occur among populations, representing local adaptation to the resource environment. For example, studies of resource polymorphisms in perch show that individuals occupying the pelagic zone are more streamlined and have a longer gut than individuals occupying the littoral zone[2,6]. Previous studies focus mainly on the influence of morphology on diet composition by phenotypic plasticity or natural selection[4,7], but the differential use of food resources could also affect other internal phenotypic traits, such as digestive performance. Gut length can exhibit phenotypic plasticity with food quality. Animals feeding on low-quality food usually have a larger digestive tract compared to animals feeding on more easily digested food resources[8]. The longer digestive tract induced by competition appears to be an adaptive response that allows more efficient digestion and more rapid growth[5]. Olsson et al.[6] reported that perch feeding on a less-digestible food type developed relatively longer guts compared to fish feeding on a more easily digested food type. Animals are expected to trade-off the size of their digestive tract between the digestive requirements of their food and the costs involved in maintaining a large organ system. Intraspecific variation in trophic morphology and resource use is extremely common in freshwater fish, but rarely is it known whether this variation represents a phenotypically plastic response to changing resource availability[1,9].

The planktivorous filter-feeding silver carp (*Hypophthalmichthys molitrix*) is one of the most intensively cultured fish species in Asia and comprises much of the production of Chinese aquaculture[10]. Recently, silver carp have been extensively cultured in lakes and reservoirs for the control of cyanobacterial blooms[11]. As a filter-feeding fish, silver carp is a generalist filter feeder that consumes many kinds of plankton, and food consumed by them strongly depends on food availability in the environment[12,13]. Previous studies mainly rely on comparisons of food habitat and resource utilization in different water bodies[14]; however, none of these studies compare resource polymorphisms of silver carp in different food-resource environments.

We examined the effect of food quality on the gut length of silver carp. We evaluated the changes of gut length in different food-resource environments and seasonal dynamics, and then tested the hypothesis that silver carp feeding on different food resources can lead to morphological trade-offs in gut length.

## METHODS

The study was carried out in Meiliang Bay of Lake Taihu, China. This region has been listed as one of the hypereutrophic parts of Lake Taihu, where heavy *Microcystis* blooms have occurred regularly, usually lasting for 8 months in a yearly cycle. From January 2004 to December 2005, silver carp and bighead carp were pen cultured in three large fish pens in an attempt to control cyanobacterial blooms in Meiliang Bay, which was part of a national restoration program for Lake Taihu. The area of each pen was 0.36 $km^2$ and the average water depth was about 2 m. The mesh size of the net was 2 × 2 cm. The fingerlings of silver and bighead carp were all purchased from a local fish hatchery. In 2004, the stocking densities of both carp were very low, with a total density of only 4.5 g $m^{-3}$ in December. During December 2004 and January 2005, we harvested all fish in the pens and restocked the fingerlings of both carp with a higher density. There was a significantly higher stocking level in 2005, with a total density of 54 g $m^{-3}$ in December. Silver carp and bighead carp, respectively, comprised about 70 and 30% of the total yield of the fish pen in 2 years.

Sampling was conducted monthly during April 2004 and October 2005. Five-to-thirty silver carp were randomly captured from the fish pens by multimesh gillnet every month. As large fish removals could influence the results in future months, we chose a relatively low sample size in each month, especially in 2004, with low stocking density. In parallel with the fish sampling, surface temperature of the lake water was measured with a thermometer. Fish specimens were placed on ice as soon as they were captured, and kept chilled or frozen until examined. In the laboratory, fish were weighed, measured, and dissected on a chilled cutting board (~ 4°C). The gut was removed and then uncoiled, without stretching, and the total gut length (GL ± 1 mm) was measured. Because silver carp have no stomach, gut length was measured from the beginning of the esophagus to the anus. Relative gut length (RGL) was calculated as follows: RGL = gut length (mm)/standard body length (mm). Relative intestinal length is the most commonly used descriptor of alimentary tract morphology in fish, particularly within families[15].

For diet analysis, five individuals were dissected at the same time, and the anterior part of the gut (pharynx – first bend) was removed and preserved in 10% formaldehyde solution. In later analysis, the gut contents were separated in cool distilled water by light agitation with a magnetic stirrer and then examined under a microscope. The food items were identified to the lowest possible taxon. Diet composition was analyzed from gut samples based on the biomass of food items. Crustacean zooplankton were examined under ×40 magnification. Biomass (wet weight) of crustacean zooplankton was estimated using the length-weight regressions according to Zhang and Huang[16]. Phytoplankton were counted and measured under ×400 magnification and rotifers were examined under ×100 magnification. To count *Microcystis* cells, the samples were agitated by gentle ultrasonication to split the colonies into single cells. Phytoplankton biovolume was estimated according to the appropriate geometric shapes, and the volume of rotifers was estimated according to the formulae given by Ruttner-Kolisko[17]. Biomass (wet weight) was calculated assuming a wet weight density of 1 g $cm^{-3}$[16]. We used *T*-test to evaluate the difference of the diet composition and RGL of silver carp between the 2 years.

## RESULTS

### Water Temperature and Diet Composition of Silver Carp

The temperature of the lake water showed quite similar seasonal dynamics in both years (Fig. 1). The highest water temperature was 33.5°C in August 2004 and 31.2°C in August 2005, respectively. In the main growth season of fish (from April to October), the average water temperature was 24.4°C in 2004 and 24.7°C in 2005, respectively. The composition of gut contents of silver carp showed great variation over time in 2 years (Fig. 2). The largest proportion of zooplankton in gut contents reached 86.6% in September 2004, whereas the least was only 8.4% in May 2005. In the main growth season (from April to October), silver carp significantly preyed on more zooplankton in 2004 (53.4% of the diet) than in 2005 (19.6% of the diet) ($p < 0.01$).

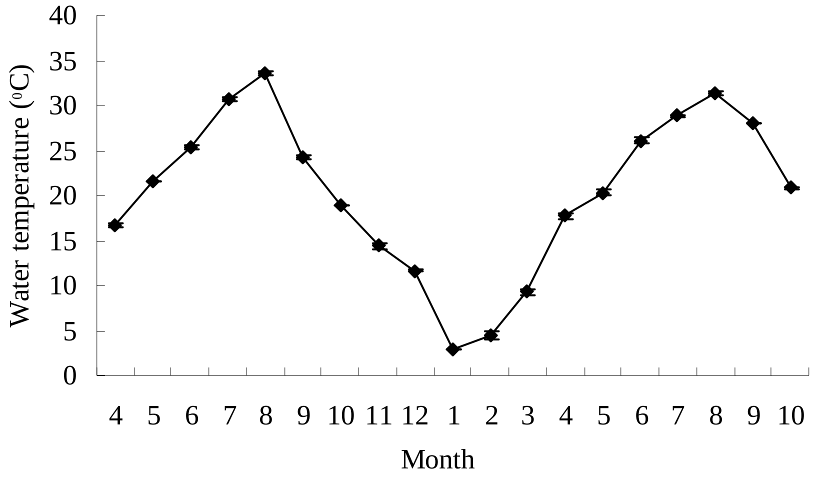

**FIGURE 1.** Temporal variations of water temperature (°C) during the study period.

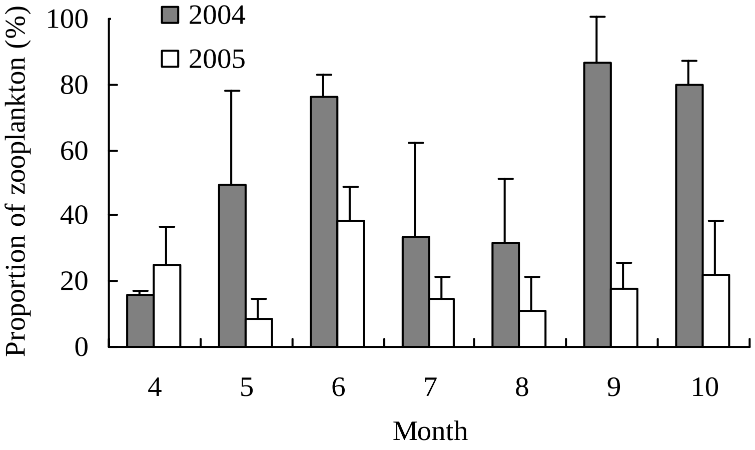

**FIGURE 2**. Comparison of zooplankton proportion in the gut contents of silver carp from April to October in 2004 and 2005. Error bars represent 1 S.D.

## Gut Length

There were significant correlations between gut length and standard body length in 2 years, and silver carp showed a longer gut in 2005 than in 2004 (Fig. 3). In the main growth months (from April to October), RGL was significantly longer in 2005 than in 2004 ($p < 0.01$, 9.23 ± 1.80 in 2004 and 10.77 ± 2.05 in 2005, respectively). RGL of silver carp varied monthly, always being longer in the summer (Fig. 4). In 2004, no correlation was found between percent zooplankton in the diet and relative intestine length, while such correlation was nearly significant in 2005 ($p = 0.112$, Fig. 5).

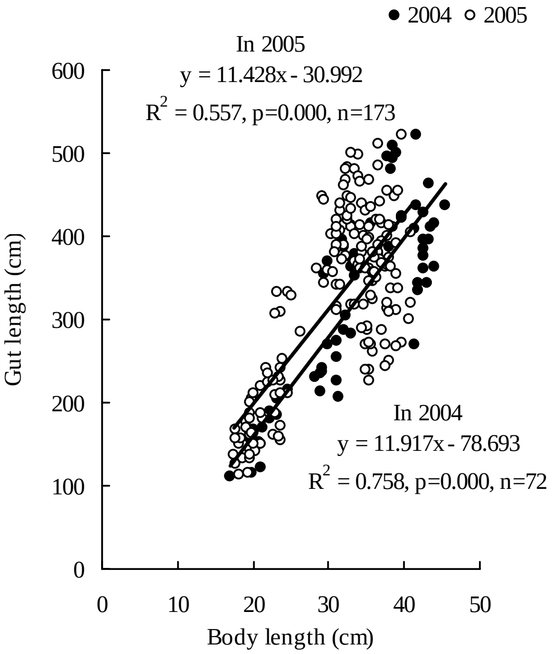

**FIGURE 3**. The relationships between the standard body length and gut length in 2004 and 2005. Open symbols and upper line represent individuals in 2005, n = 173, whereas solid symbols and nether line represent individuals in 2004, n = 72.

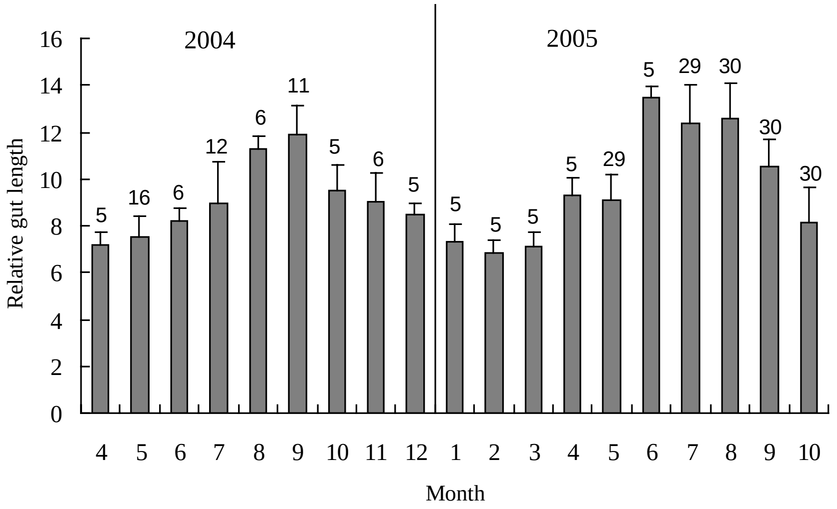

**FIGURE 4.** Seasonal changes of RGL of silver carp during April 2004 and October 2005. Error bars represent 1 S.D. Values above the bars indicate the number of samples.

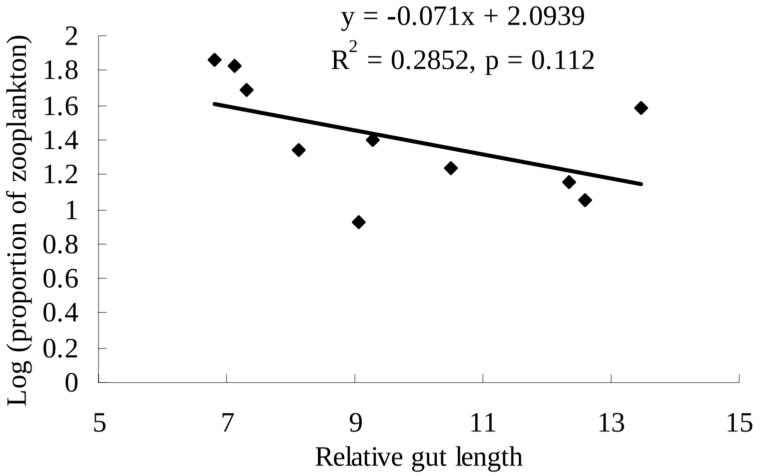

**FIGURE 5.** The relationships between RGL of silver carp and proportion of zooplankton in the diet from January to October in 2005.

## DISCUSSION

Analysis of gut contents showed that silver carp fed on more zooplankton under a low stocking density in 2004. Stocking density is known to have a high impact on the use of resources[18]. The size and density of food resources can exert great influence on the feeding habits of most filter-feeding fish[13,14]. In comparison with zooplankton, phytoplankton are low in energy and other nutrients, and are difficult to be digested by fish due to their strong cell walls and high content of indigestible material[19]. Krebs[20] reports that predators can maximize the overall rate of uptake of nutrients if they eat the most profitable food and tend to ignore less-valuable food when more profitable ones are abundant. Our results suggest that in a resource-rich environment, silver carp increased their energy intake through feeding on more zooplankton.

Plasticity in the digestive organs is expected among animals experiencing temporal or spatial differences in food quality[6,21]. Magnan and Stevens[22] found that brook charr (*Salvelinus fontinalis*)

feeding on their less-preferred diet, zooplankton, had longer pyloric ceca compared to charr feeding on their preferred diet of macroinvertebrates. Pumpkinseed sunfish (*Lepomis gibbosus*) can exhibit considerable intraspecific variation in pharyngeal jaw morphology by raising them on diets with or without snails[4]. Hjelm et al.[23] reported that young-of-the-year perch (*Perca fluviatilis*) display rapid plastic responses in body morphology to different environmental conditions. Many previous studies relating to resource polymorphisms focus mainly on external morphology, but the differential use of food resources could also influence other internal phenotypic traits, such as the digestive performance[6]. In the present study, we found that silver carp had a longer gut when feeding on more phytoplankton. Evolutionary theory predicts that individuals should develop organs that meet the functional demand that is placed on them[8,24]. For example, several bird species can increase their gut lengths in response to increased dietary fiber content and increased intake[25]. Optimal digestion theory suggests a tight link between the morphology of the digestive system and food quality[8]. Our results corroborate these predictions. Silver carp adjusted their gut length to match the digestive requirements of the food when being exposed to a different food resource. In 2005, the gut length of silver carp increased in response to a decline of the nutrient-rich and easily digestible zooplankton in their food items, indicating a functional trade-off between performance in alternate food resource and mass of digestive tissue. Longer digestive systems allow for longer transit times and increase the efficiency of nutrient extraction from food. Such a trade-off in gut length suggests importance for the best use of resources, especially under high competition.

We found an obvious seasonal variation of gut length in silver carp. Seasonal differences in gut length might be the result of ontogenetic or environmental variation acting on a single plastic genotype[2,4]. Our results suggest that seasonal changes of RGL of silver carp might be an adaptive response to the variability of food digestibility. In the present study, there was a significant negative correlation between RGL and phytoplankton contribution in diet in 2005, and RGL increased when silver carp was forced to feed on more phytoplankton. Moreover, our results suggest that silver carp were able to respond rapidly to changes in diet quality by altering gut length, especially under high competition, while in the low stocking density, there was no significant correlation between gut length and food quality. Relyea and Auld[5] report that intra- and interspecific competition can induce relatively longer gut, and that competition-induced gut plasticity may be quite common in tadpoles. Battley and Piersma[25] indicate that an increase of intake is also an important factor leading to an increase in gut length in birds, suggesting that food quality may not be the only selective force acting on gut length. Except for food quality, we extrapolate that other ecological conditions (such as competition or feeding intensity) could also influence gut morphology. In the present study, it seems that the relationship of gut length and food quality is more easily masked by other environmental factors in low competition.

The present study suggests that silver carp might have evolved plasticity to change their gut length rapidly to facilitate efficient utilization of food resource. Such plasticity may increase fitness when high-quality foods are unavailable or when food quality changes rapidly. Phenotypic plasticity represents an important mechanism that can mold a fish’s morphology to the resource base of a lake. Our work provided field evidence for understanding the functional basis of resource polymorphisms and the evolution of phenotypic plasticity in planktivorous filter-feeding fishes.

## ACKNOWLEDGMENTS

We would like to express our sincere thanks to Dr. Allen and two anonymous reviewers for their useful comments and suggestions on the manuscript. This research was supported by an 863 project titled “Study on Eco-techniques for Improving Water Quality of Drinking Water Source in Meiliang Bay, Lake Taihu” (Grant No. 2002AA601011).
